# Case Report: COVID-19 Pandemic Exacerbates Eating Disorder by Social and Intrafamilial Isolation

**DOI:** 10.3389/fped.2022.819214

**Published:** 2022-02-24

**Authors:** Yoshiki Katsumi, Kazuki Kodo, Sachiko Goto

**Affiliations:** Department of Pediatrics, Saiseikai Kyoto Hospital, Nagaokakyo, Japan

**Keywords:** COVID-19, feeding and eating disorders, intrafamilial isolation, stress, weight loss

## Abstract

The coronavirus disease-2019 (COVID-19) pandemic has increased the stress levels of children and their parents and diagnoses of eating disorders (EDs), irritable bowel syndrome, migraines, tension headaches, orthostatic dysregulation, and/or school refusal has increased among children. We present a case of a nine-year old girl, which rapidly worsened due to stress and isolation related to the COVID-19 pandemic. The patient's father noted her rapid weight loss due to poor oral intake. While she had already stopped gaining weight before the pandemic, her weight rapidly decreased to 22 kg during the pandemic. We diagnosed her with an ED and administrated nasogastric tube feeding. We postulated that not only social isolation, but also the disruption in her relationship with her parents, due to the pandemic, contributed to her ED. During a family meeting, she revealed that she felt more anxious during the pandemic. After the meeting, her parents rescheduled their jobs so that the family can have dinner together every night. The patient started eating sufficiently and weighed 31.8 kg at the one-year follow-up. The proportion of children with ED increased during the pandemic; their symptoms worsened because they felt lonely due to social and intrafamilial isolation. While parents have themselves experienced more stress during the pandemic, children, including those with ED, have experienced increased stress related directly to the pandemic, as well as indirectly from their parents. Pediatricians should consider the impact of stress on children, especially from social and intrafamilial isolation, both during and after the pandemic.

## Introduction

During the coronavirus disease-2019 (COVID-19) pandemic, children and their parents experienced high levels of psychological stress, even if they were not infected with the coronavirus (1, 2). The proportion of children with eating disorders (ED) increased during the pandemic due to social isolation and closure of schools, resulting in disruption of protective factors, such as extra-curricular activities, school routine, and peer relationships (3). In Japan, even during the pandemic, there are families in which both parents must work daily; therefore, in many cases, the children stay at home alone when schools are closed. This causes a disruption of familial relationships, leading to increased ED in children. We present a patient with an ED that was rapidly exacerbated due to stress and environmental change during the pandemic; the patient experienced marked improvement in both intrafamilial relationship and symptoms after a family meeting.

## Case Presentation

A 9-year-old girl was brought to the hospital by her father because of poor oral intake and rapid weight loss within a few months along with significant irritability during the day. Her family included her parents, a 6-year-old sister, and a 2-year-old brother. She had no history of disease which affected her feeding. There was no other identifiable cause of weight loss, such as unavailability of food at home or lack of interest in eating. She was neither involved in excessive exercise nor exposed to social media. She attended after-school English and piano classes, and also attended the Kumon Math Program classes 4 days a week. She weighed 22 kg, with a body mass index of 12.0 kg/m^2^. She presented with bradycardia (heart rate: 40 beats per minute) and hypotension (82/56 mm Hg). Her skin was dry, and the capillary refill time was 3 secs, indicating dehydration. Blood tests revealed low levels of blood sugar (49 mg/dL), pre-albumin (10.1 mg/dL), transferrin (148 mg/dL), alkaline phosphatase (215 U/L), insulin-like growth factor-1 (17 ng/dL), and free triiodothyronine (1.3 pg/mL), which resulted from chronic poor oral intake. The patient was admitted to manage her health problems and to improve her eating habits.

**Figure 1:**
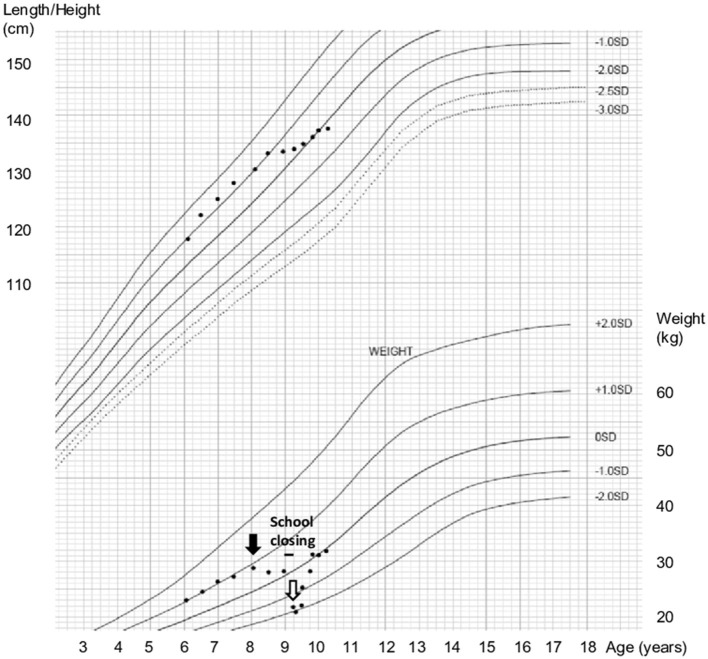
Growth stopped before the COVID-2019 pandemic and weight loss began during the pandemic. Growth stopped a year and 3 months before admission (black arrow). School had been closed for over 3 months (black bar). She was admitted due to rapid weight loss (white arrow).

We plotted her growth curve and found that she had stopped gaining weight 15 months earlier. Furthermore, when schools were closed due to the COVID-19 pandemic, she lost approximately 6 kg of weight rapidly in the next 3 months (Figure 1). The child scored 16 (below cut-off of 18) on the Eating Attitudes Test 26, although questions on social pressure to eat were highly scored. She exhibited no preoccupation for thinness or excessive physical activity. Thus, she was diagnosed with avoidant/restrictive food intake disorder, according to the criteria of Diagnostic and Statistical Manual of Mental Disorders.

After admission, intravenous hydration (30 ml/day of Solita-T3^®^, Otsuka, Japan) for 2 weeks and oral nutritional supplements (250 ml/day of Meibalance^®^, Meiji, Japan, or 500 ml/day of Ensure^®^, Abbott, Japan) for 1 week, both of which were not enough for her to gain her weight, were administered. However, her weight decreased to 21 kg because she was not eating. Nasogastric tube feeding was required temporarily. A family meeting was organized to help her express her feelings, needs, and wants. During the meeting, she shared that she felt anxious whenever her mother left the house for work, and that her anxiety increased during the pandemic. However, she tried to be patient and was not willing to tell her parents about it for a long time. After the meeting, she felt better, and her parents rescheduled their working hours to improve her health. The patient started eating sufficiently and her weight increased gradually. She was discharged after 2 months of admission. We carefully followed up by checking her body weight and assessing her relationship with her parents. She weighed 31.8 kg at the one-year follow-up visit. The patient and her parents provided informed assent, and this case report was approved by the appropriate ethics review board.

## Discussion

Our patient had an ED for over 1 year before the pandemic. Her growth stopped when her mother resumed work after her younger brother turned one, and she started after-school activities, such as English classes, piano lessons, and the Kumon Math Program classes. She probably endured the shallow relationship with her parents and maintained her self-esteem by studying hard. However, when the government declared a state of emergency in Japan, she could not continue with school and after-school activities. Following this, her ED rapidly exacerbated. She had to stay at home and take care of her 6-year-old sister when her parents left for work daily. In addition, her parents complained about each other and argued in front of her. Her parents had to work hard and stay out of home every day during the pandemic, and they could not take off time to take care of her when she was lacking appetite and showing other signs of her disorder. Furthermore, her schoolteachers did not notice her weight loss when school reopened because her home teacher was changed at the beginning of the school year. Her ED rapidly worsened due to stress caused by the social and intrafamilial isolation as well as the many complaints and arguments between her parents. Therefore, we can say that multiple factors are involved in the exacerbation of ED in children during the pandemic.

**Figure 2:**
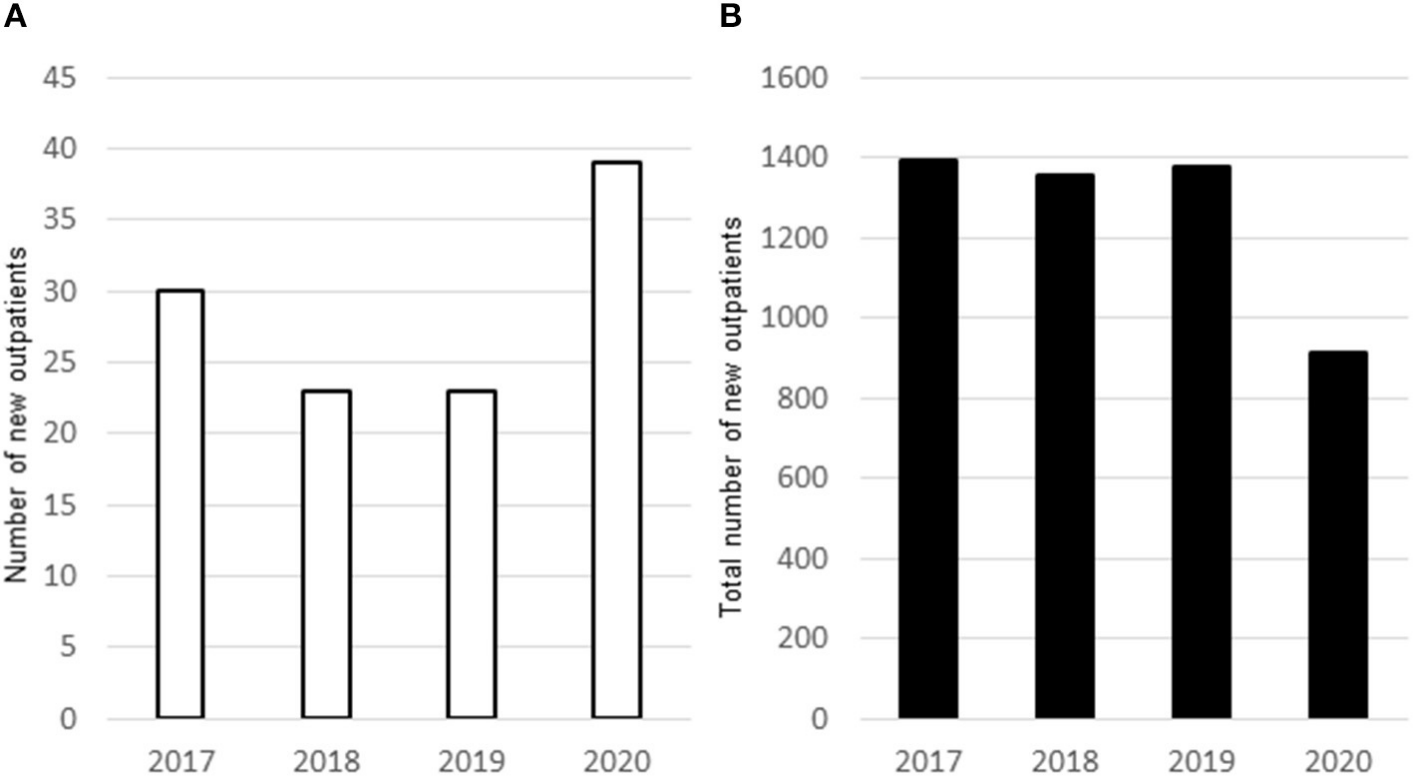
In 2020 with the COVID-2019 pandemic, there was an increase in newly diagnosed outpatients (age >16 years) with eating disorders, irritable bowel syndrome, migraine, tension headache, orthostatic dysregulation, and/or school refusal, **(A)**. The number of new outpatients (age >16 years) decreased in at Saiseikai Kyoto Hospital **(B)**.

One of the causes of ED in children is increased stress in children and their parents. According to a study, parents' stress increased from even before the pandemic to May 2020 (at the peak of mandatory staying at home) and remained elevated above pre-COVID-19 values in September 2020 (children's return to school) (1). Parents suffered from a shortage of relaxation time, difficulty in child rearing, increased partner aggression, and an increased sense of unfairness during the pandemic (2). These two studies have no data on ED in children; however, the authors suggest that children (including those with EDs) experienced stress directly due to the COVID-19 pandemic and indirectly due to their parents. In this case, the children, including our patient, were left alone at home as the parents had to go to work every day and needed to be careful to prevent coronavirus infection at their workplaces. The mother also complained about unfair division of labor to the father during the pandemic. All these factors increased the stress of the children and the parents which led to the worsening of symptoms of ED in the patient. Furthermore, the number of patients with EDs that were under 16 years of age, who required admission due to worsening symptoms, increased during the pandemic (3). We noticed that the number of new outpatient cases (under 16 years of age) who presented to our department with ED, irritable bowel syndrome, migraines, tension headaches, orthostatic dysregulation, and/or school refusal, which could be caused by stress, increased in 2020; conversely, the total number of new outpatient cases of different diseases, particularly infectious diseases (which accounted for most outpatient cases before the pandemic), markedly decreased in the same age group (Figure 2). This marked decrease in the number of new infectious disease cases could be explained by improvement in hygiene methods and practices during and after the COVID-19 pandemic.

In this case, the family meeting helped to improve the patient's symptoms. She obviously did not spend enough time with her parents during the pandemic; therefore, we can say that intrafamilial isolation had a greater effect on her ED than social isolation. We suggested that her parents should reschedule their work and have dinners together every day. This is a simple but particularly important strategy for patients with ED, especially cases with intrafamilial isolation. Family meal session has been recently used as a strategy for ED (4). Having dinner with family is a natural thing, but pediatricians are frequently consulted by patients with ED, who feel lonely because they do not have dinner with their parents. Therefore, physicians should ascertain whether patients with ED have enough time with their family during the pandemic.

If the parents can reschedule their work, family meetings may be particularly effective for children who do not spend much time with their parents, and therefore feel lonely. Flexible working results in improved employee mental health (5). Parents with mental health problems may indirectly affect ED symptoms in pediatric patients (6). In our case, we asked the patient's mother's employer to reduce her work hours to make time for family dinners. The workload reduction probably improved the mother's mental health and resulted in successful family meetings.

There are some limitations to the findings of this report. The family meeting may not have been the only cause for the patient's recovery. We regularly checked the relationship between her and her family after the meeting, but the feeding and weight gain may have resulted from self-recovery or our sustained attention over time.

The World Health Organization and the Japanese government have suggested that people should adjust to the “new normal” even after the pandemic (7, 8); therefore, children and adults may remain under stress for the next few years. Therefore, pediatricians should be aware that children may be experiencing great stress, especially from social and intrafamilial isolation, during and after the pandemic.

## Data Availability Statement

The original contributions presented in the study are included in the article/supplementary material, further inquiries can be directed to the corresponding author.

## Ethics Statement

The studies involving human participants were reviewed and approved by the Ethics Committee of Saiseikai Kyoto Hospital. Written informed consent to participate in this study was provided by the participants' legal guardian/next of kin. Written informed consent was obtained from the individual(s), and minor(s)' legal guardian/next of kin, for the publication of any potentially identifiable images or data included in this article.

## Author Contributions

YK was the patient's main healthcare provider, drafted the initial manuscript, reviewed, and revised the manuscript. KK and SG assisted with clinical treatment and reviewed and revised the manuscript. All authors have read and approved the final manuscript.

## Conflict of Interest

The authors declare that the research was conducted in the absence of any commercial or financial relationships that could be construed as a potential conflict of interest.

## Publisher's Note

All claims expressed in this article are solely those of the authors and do not necessarily represent those of their affiliated organizations, or those of the publisher, the editors and the reviewers. Any product that may be evaluated in this article, or claim that may be made by its manufacturer, is not guaranteed or endorsed by the publisher.

## References

[1] Adams EL Smith D Caccavale LJ Bean MK. Parents are stressed! patterns of parent stress across COVID-19. Front Psychiatry. (2021) 12:626456. 10.3389/fpsyt.2021.626456 33897489 PMC8060456

[2] Kimura M Kimura K Ojima T. Relationships between changes due to COVID-19 pandemic and the depressive and anxiety symptoms among mothers of infants and/or preschoolers: a prospective follow-up study from pre-COVID-19 Japan. BMJ Open. (2021) 11:e044826. 10.1136/bmjopen-2020-044826 33622953 PMC7907626

[3] Haripersad YV Kannegiesser-Bailey M Morton K Skeldon S Shipton N Edwards K. Outbreak of anorexia nervosa admissions during the COVID-19 pandemic. Arch Dis Child. (2021) 106:e15. 10.1136/archdischild-2020-319868 32709684

[4] White HJ Haycraft E Madden S Rhodes P Miskovic-Wheatley J Wallis A. Parental strategies used in the family meal session of family-based treatment for adolescent anorexia nervosa: links with treatment outcomes. Int J Eat Disord. (2017) 50:433–6. 10.1002/eat.22647 28393398

[5] Li LZ Wang S. Do work-family initiatives improve employee mental health? longitudinal evidence from a nationally representative cohort. J Affect Disord. (2022) 297:407–14. 10.1016/j.jad.2021.10.112 34718041

[6] Li LZ Bian JY Wang S. Moving beyond family: unequal burden across mental health patients'social networks. Qual Life Res. (2021) 30:1873–9. 10.1007/s11136-021-02782-9 33566303

[7] World Health Organization. From the “new normal” to a “new future”: A sustainable response to COVID-19. (2020). Available online at: https://www.who.int/westernpacific/news/commentaries/detail-hq/from-the-new-normal-to-a-new-future-a-sustainable-response-to-covid-19/ (accessed October 24, 2021).

[8] Japan Times. The New Normal That Will Arise After COVID-19. (2020). Available online at: https://www.japantimes.co.jp/opinion/2020/11/22/commentary/japan-commentary/new-normal-coronavirus/ (accessed October 24, 2021).

